# Supplementary figures and images for: Scalable eQTL mapping using single-nucleus RNA-sequencing of recombined gametes from a small number of individuals
Source: PLoS Biol. 2025 Apr 25;23(4):e3003085. doi: 10.1371/journal.pbio.3003085 (PMC12119024; doi:10.1371/journal.pbio.3003085)

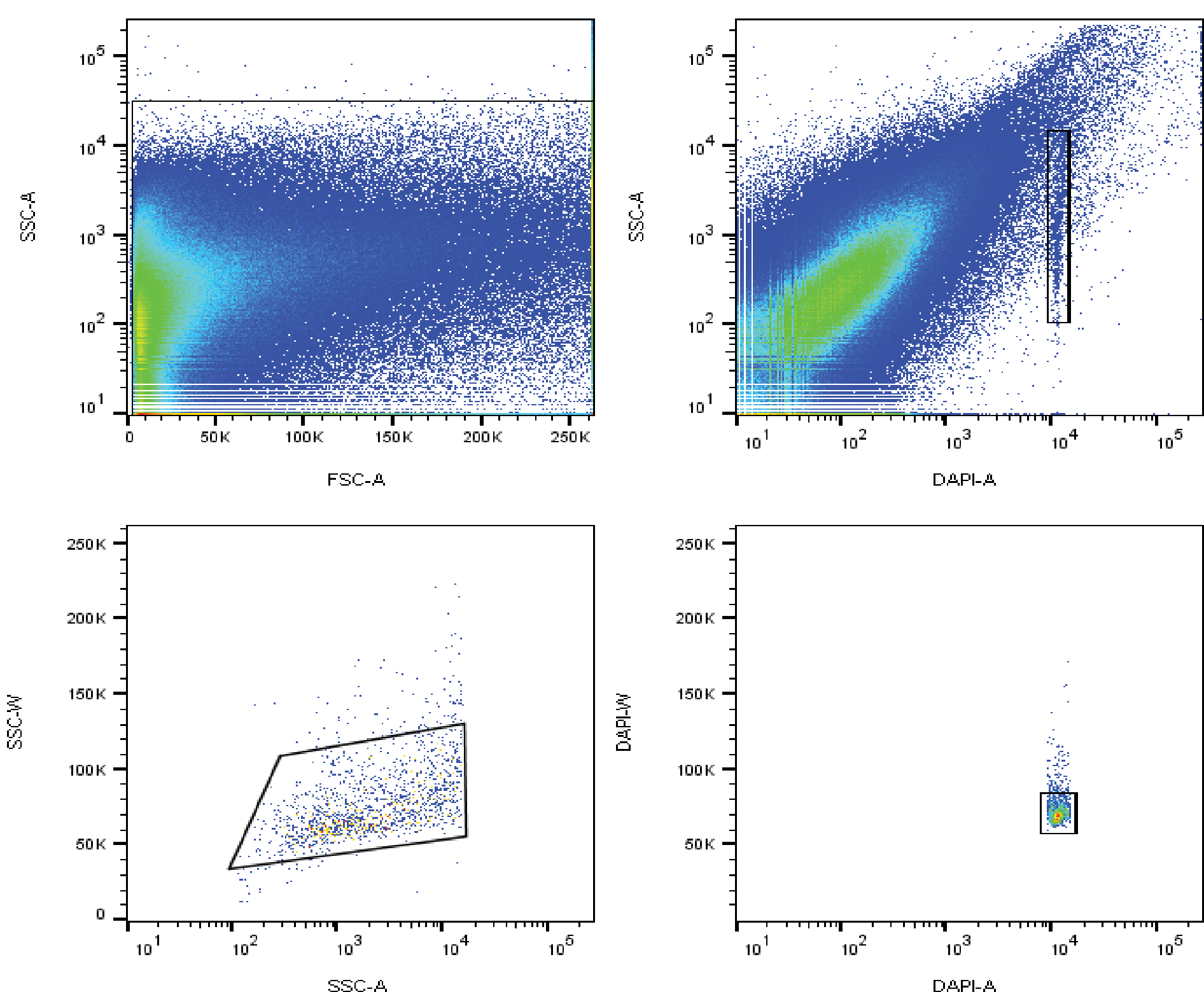

Supplement: S1 Fig — (TIFF) [file pbio.3003085.s002.tiff]

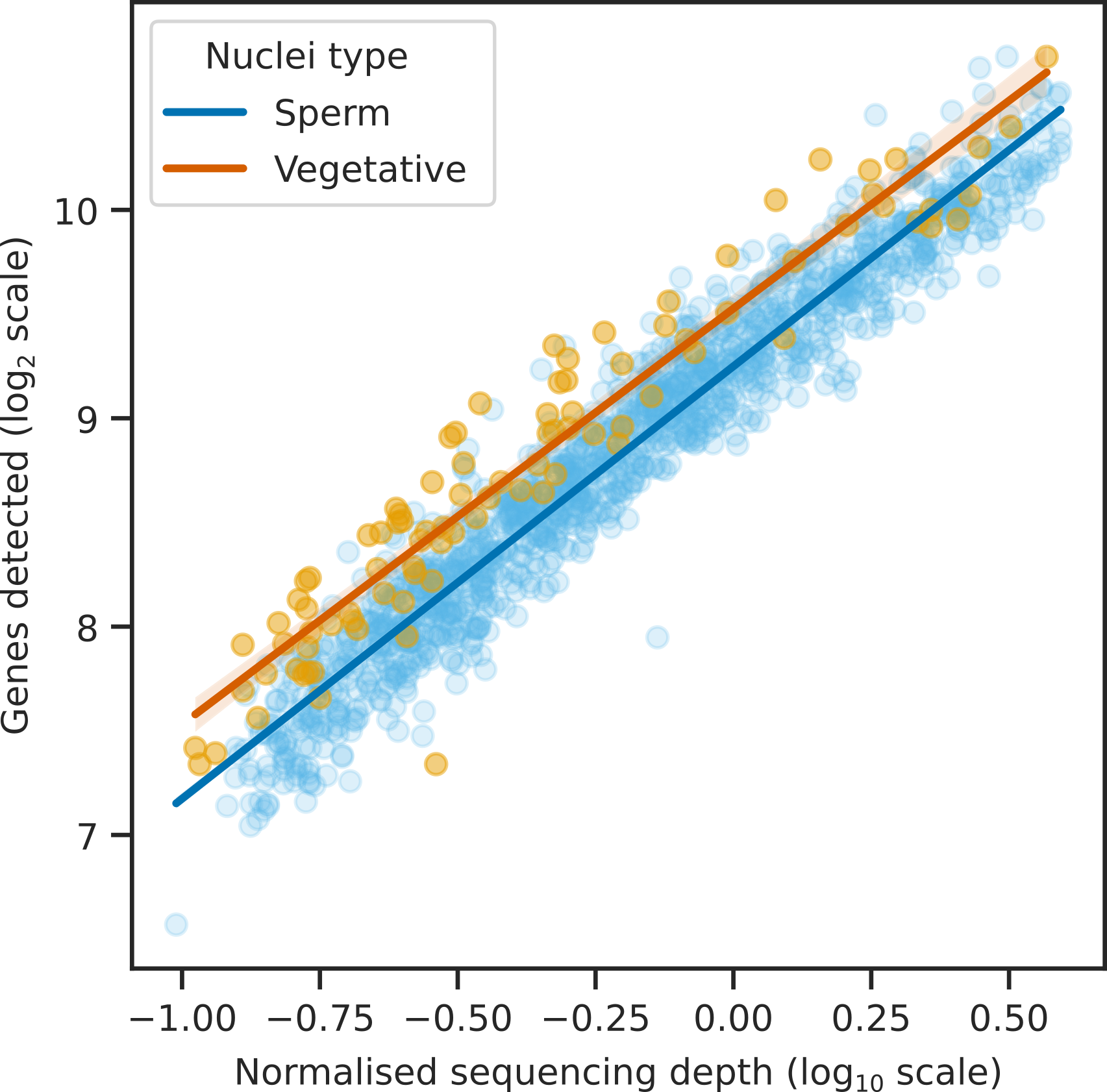

Supplement: S2 Fig — For a given sequencing depth, more genes are detected per vegetative nucleus than per sperm nucleus, demonstrating the greater transcriptomic diversity of vegetative nuclei. The data underlying this figure can be found in dataset 1 at https://doi.org/10.5281/zenodo.14864053. (TIFF) [file pbio.3003085.s003.tiff]

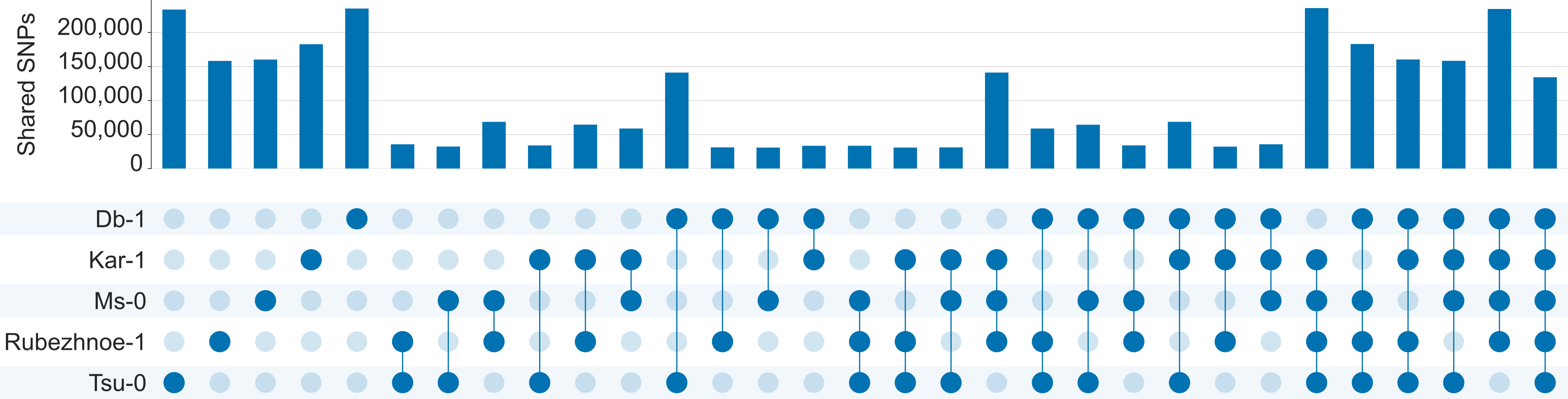

Supplement: S3 Fig — (TIFF) [file pbio.3003085.s004.tiff]

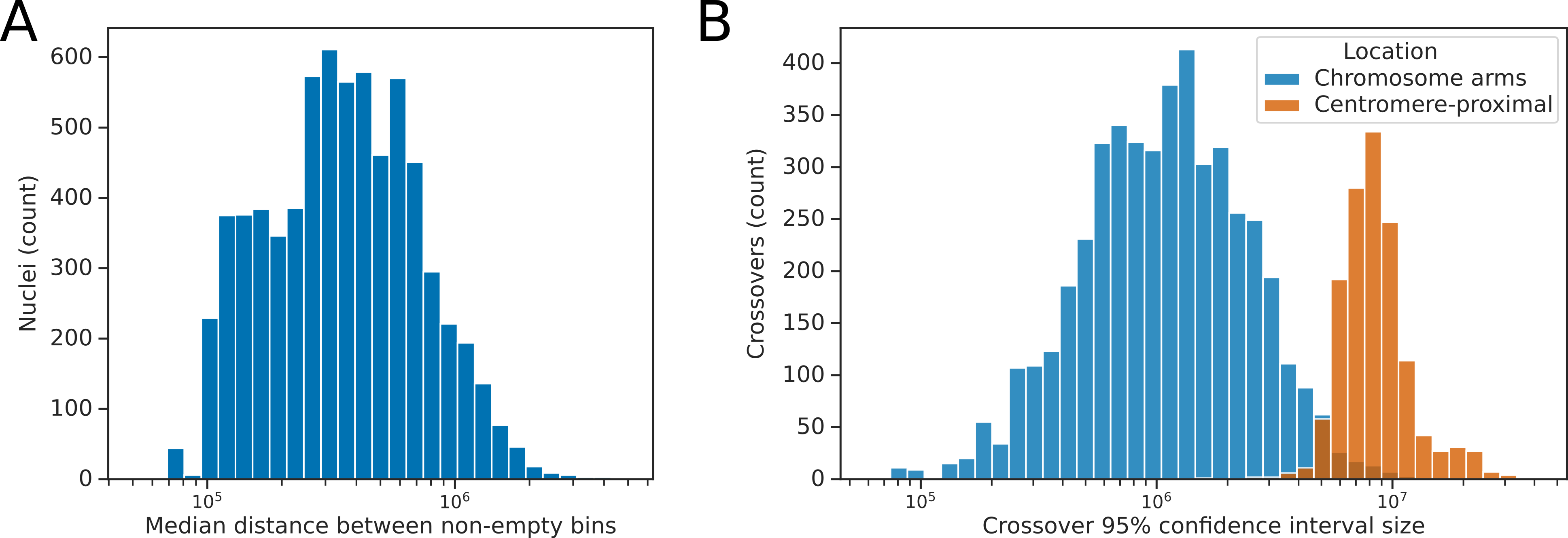

Supplement: S4 Fig — (A) Histogram showing the median distance between 25 kb genomic bins that contain at least one or more informative reads for each nucleus barcode. (B) Histograms showing the 95% confidence intervals of the positions of crossovers predicted by the rigid hidden Markov model. Crossovers called in chromosome arms (in blue) were mapped with much greater resolution than crossovers that were close to the centromere (in orange). The data underlying this figure can be found in dataset 2 at https://doi.org/10.5281/zenodo.14864053. (TIFF) [file pbio.3003085.s005.tiff]

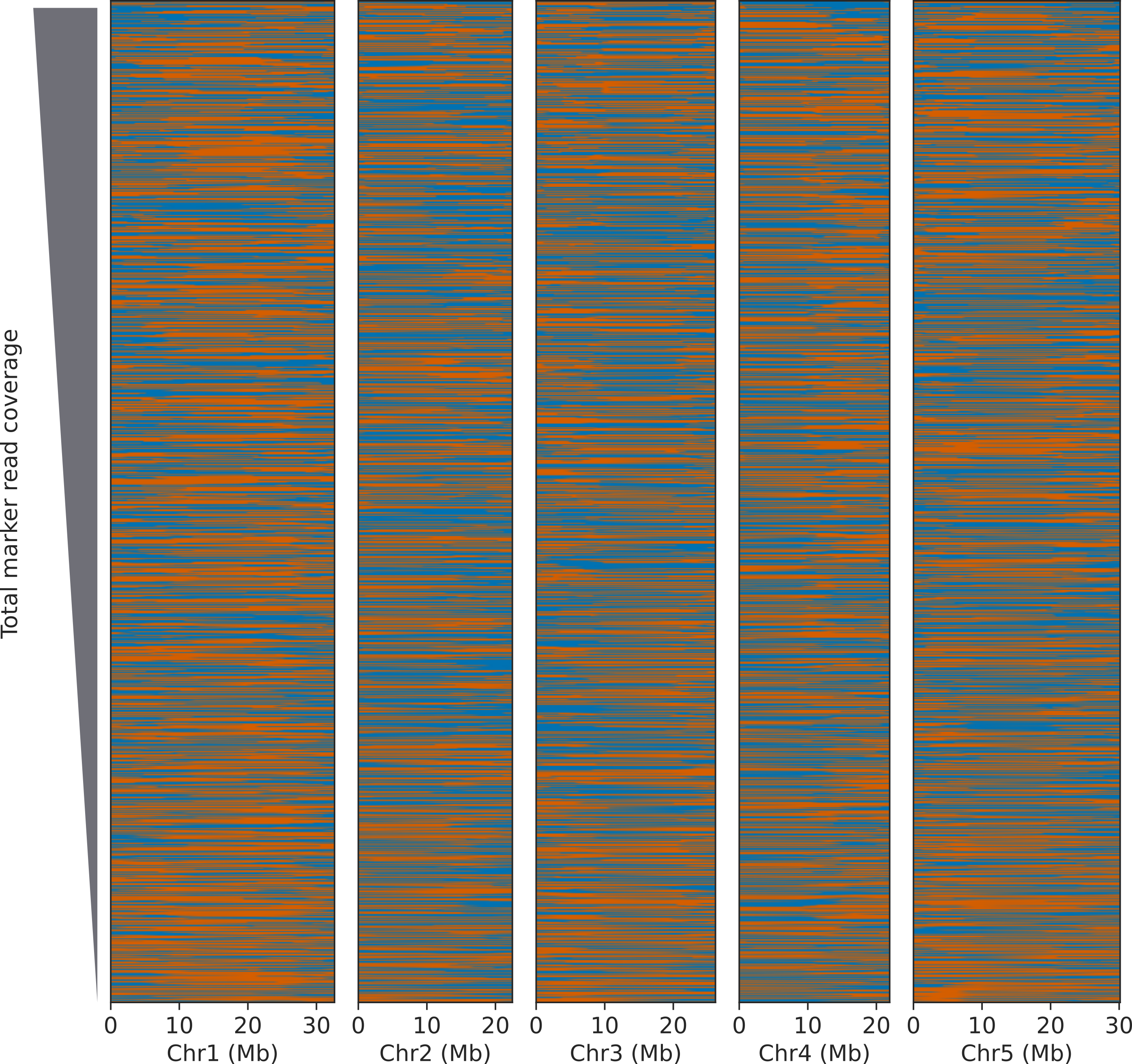

Supplement: S5 Fig — The data underlying this figure can be found in dataset 2 at https://doi.org/10.5281/zenodo.14864053. (TIFF) [file pbio.3003085.s006.tiff]

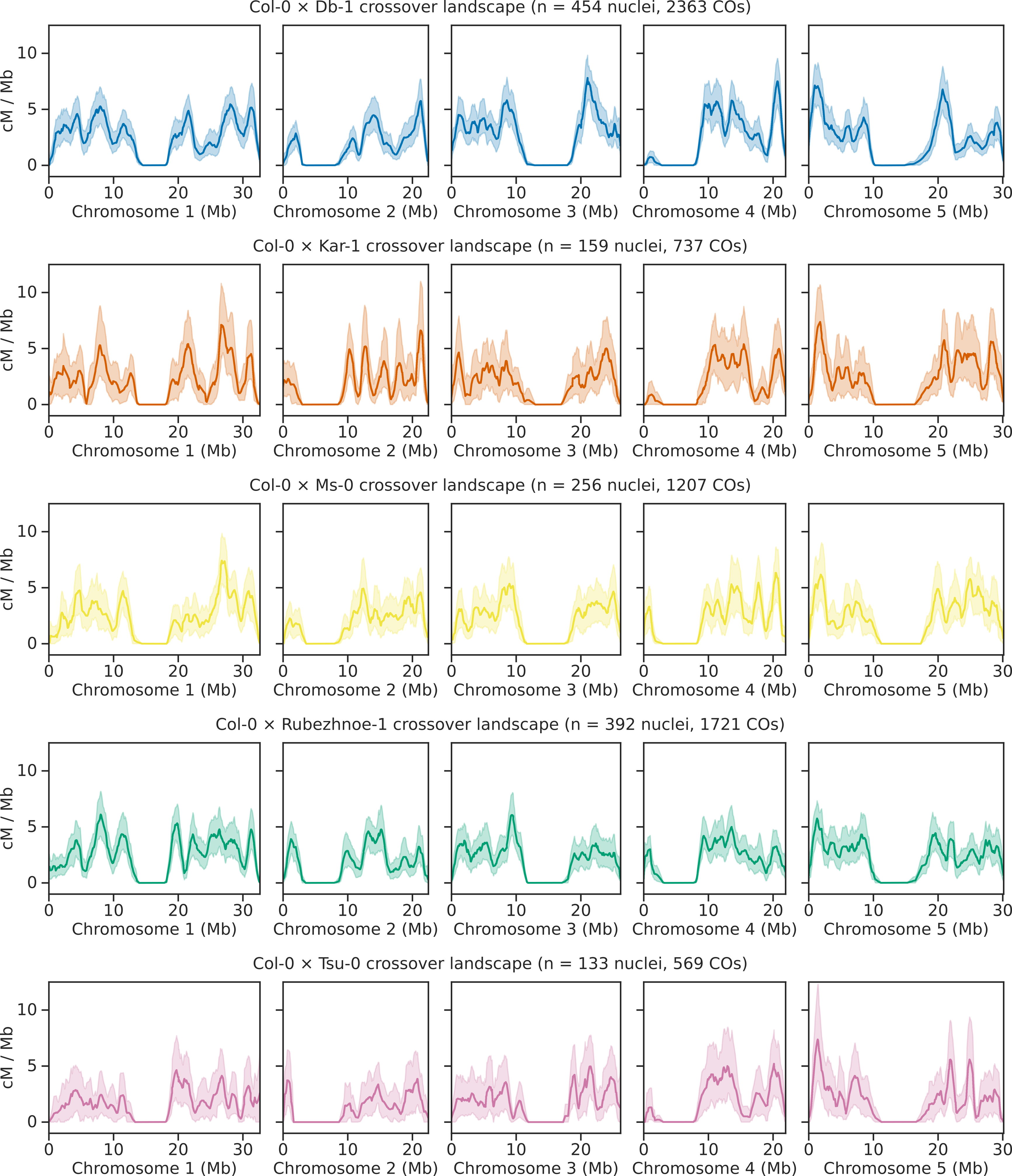

Supplement: S6 Fig — Shaded areas represent the 95% confidence intervals of the cM/Mb estimates generated using 100 bootstrapped resamples of the nuclei. The data underlying this figure can be found in dataset 2 at https://doi.org/10.5281/zenodo.14864053. (TIFF) [file pbio.3003085.s007.tiff]

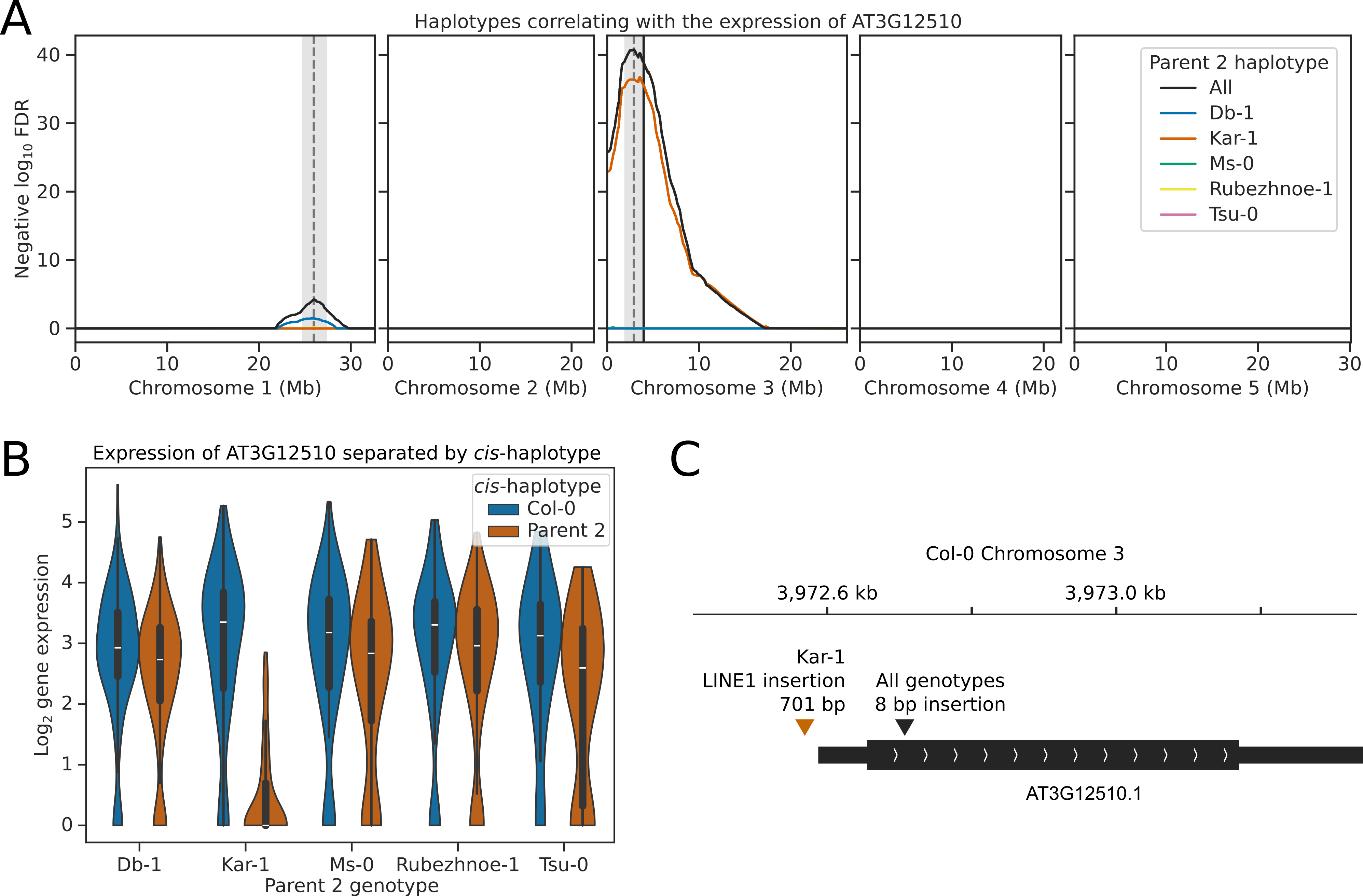

Supplement: S7 Fig — (A) eQTL plot showing the haplotypes whose inheritance correlates with the expression of the MADS-box transcription factor AT3G12510. eQTL peaks are shown as vertical dashed lines with 1.5 LOD drop confidence intervals shown as grey shaded regions. The location of the AT3G12510 gene is shown as a solid vertical black line. The black line labelled “All” shows the FDR calculated from the log ratio test of all 5 parent 2 haplotypes compared to Col-0. (B) Violinplot showing the gene expression of AT3G12510 in nuclei separated by the cis-haplotype (i.e., the haplotype at AT3G12510). Nuclei that inherit the Kar-1 haplotype of AT3G12510 have significantly reduced expression of AT3G12510, compared to sister nuclei that inherit the Col-0 haplotype. (C) The AT3G12510 gene model, showing major insertions compared to Col-0. In Kar-1, a LINE1 retrotransposable element is inserted into the AT3G12510 promoter, likely explaining the reduced expression of the AT3G12510 gene. The data underlying this figure can be found in datasets 1, 2 and 3 at https://doi.org/10.5281/zenodo.14864053. (TIFF) [file pbio.3003085.s008.tiff]

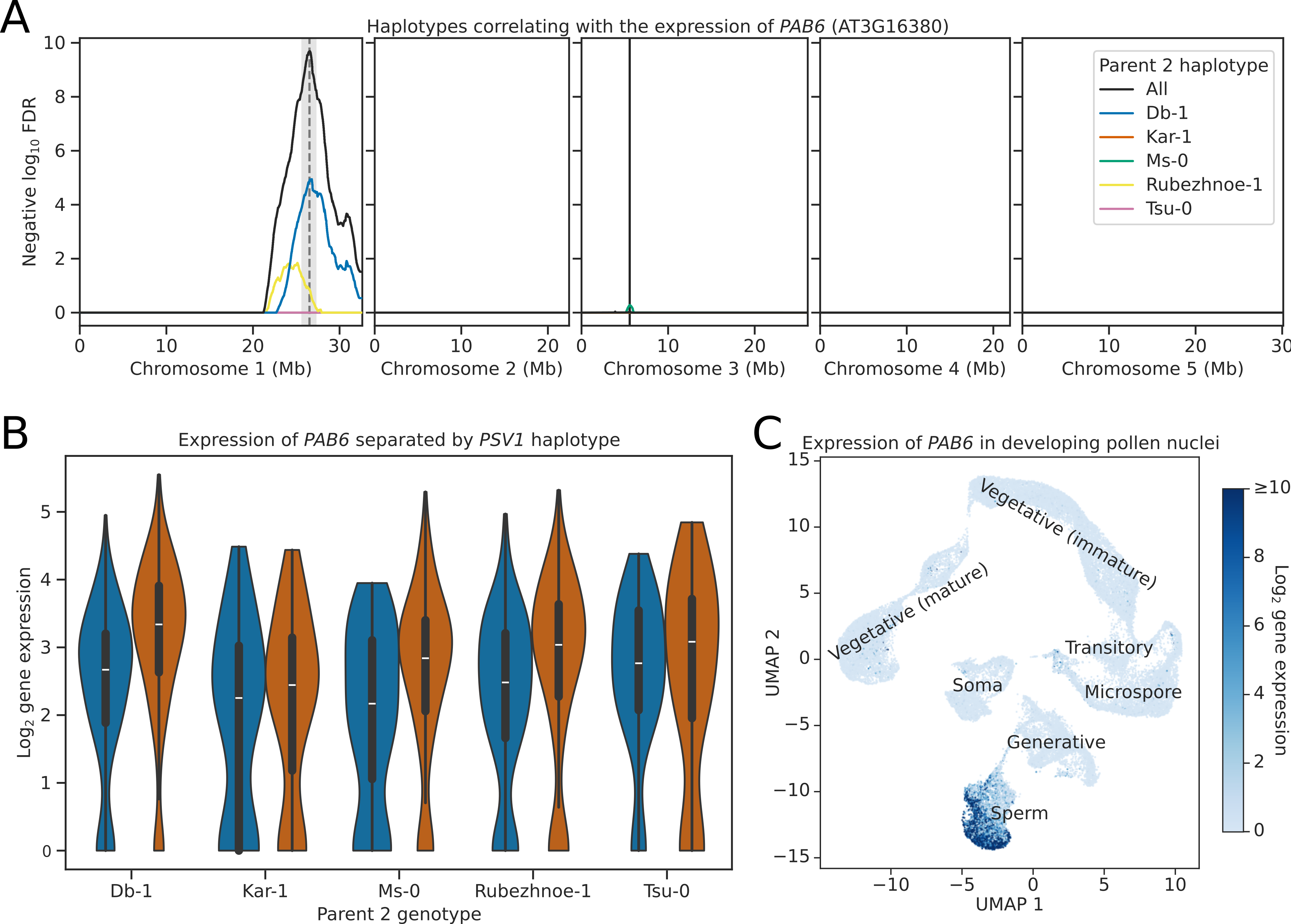

Supplement: S8 Fig — (A) eQTL plot showing the haplotypes whose inheritance correlates with the expression of the poly(A) binding protein gene PAB6 (AT3G16380). eQTL peaks are shown as vertical dashed lines with 1.5 LOD drop confidence intervals shown as grey shaded regions. The location of the PAB6 gene is shown as a solid vertical black line. The black line labelled “All” shows the FDR calculated from the log ratio test of all 5 parent 2 haplotypes compared to Col-0. (B) Violinplot showing the gene expression of PAB6 in nuclei separated by the haplotype of PSV1. Nuclei that inherit the Db-1 or Rubezhnoe-1 haplotype of PSV1 have significantly increased expression of PAB6, compared to sister nuclei that inherit the Col-0 haplotype. (C) UMAP projection from Ichino and colleagues 2022, showing the expression of PAB6 throughout the developmental stages of the pollen. PAB6 is only expressed in the sperm nucleus cluster, and is absent from microspore, generative and vegetative nuclei, as well as from the soma. The data underlying this figure can be found in datasets 1, 2 and 3 at https://doi.org/10.5281/zenodo.14864053. (TIFF) [file pbio.3003085.s009.tiff]

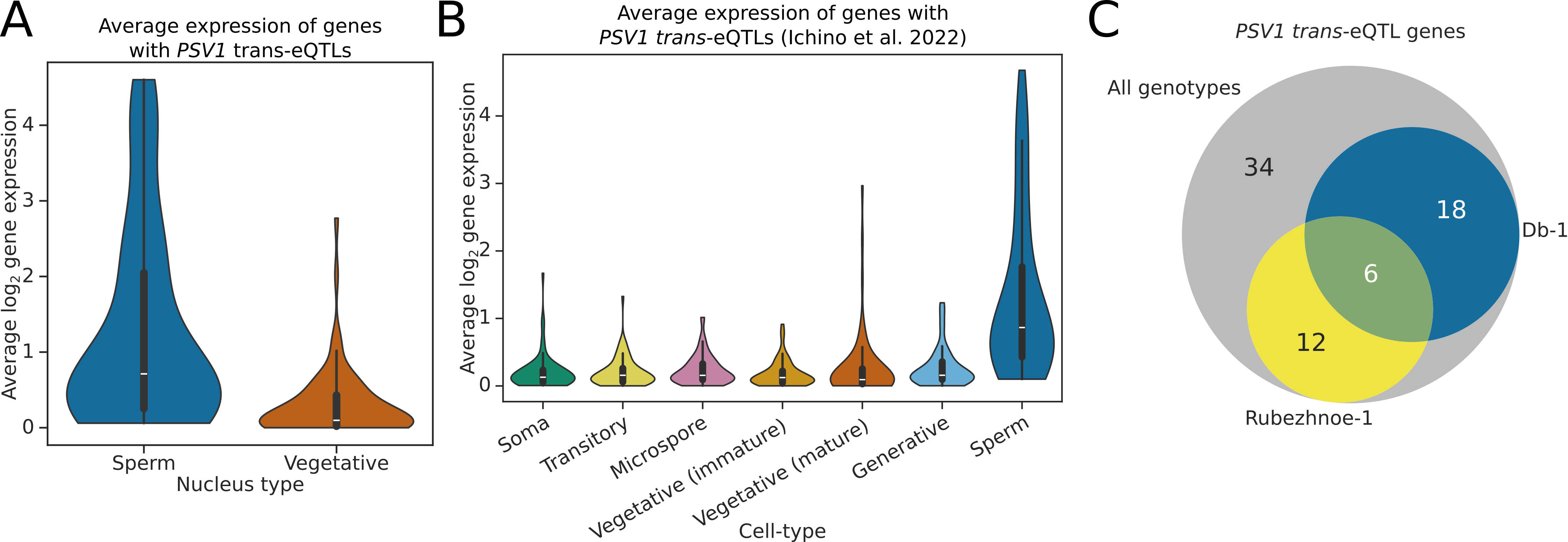

Supplement: S9 Fig — (A) Violinplot showing the average expression of genes with an eQTL peak at the PSV1 locus in sperm and vegetative nuclei. Genes affected by PSV1 tend to have greater expression in the sperm nuclei. (B) Violinplot showing the average expression of genes with an eQTL peak at the PSV1 locus in various immature and mature pollen cell-types, identified in a snRNA-seq dataset from Ichino and colleagues 2022. (C) Venn-diagram showing the overlap of PSV1 trans-eQTLs that are significant in all genotypes combined, versus in Db-1 and Rubezhnoe-1 specifically. The data underlying this figure can be found in datasets 1 and 3 at https://doi.org/10.5281/zenodo.14864053. (TIFF) [file pbio.3003085.s010.tiff]

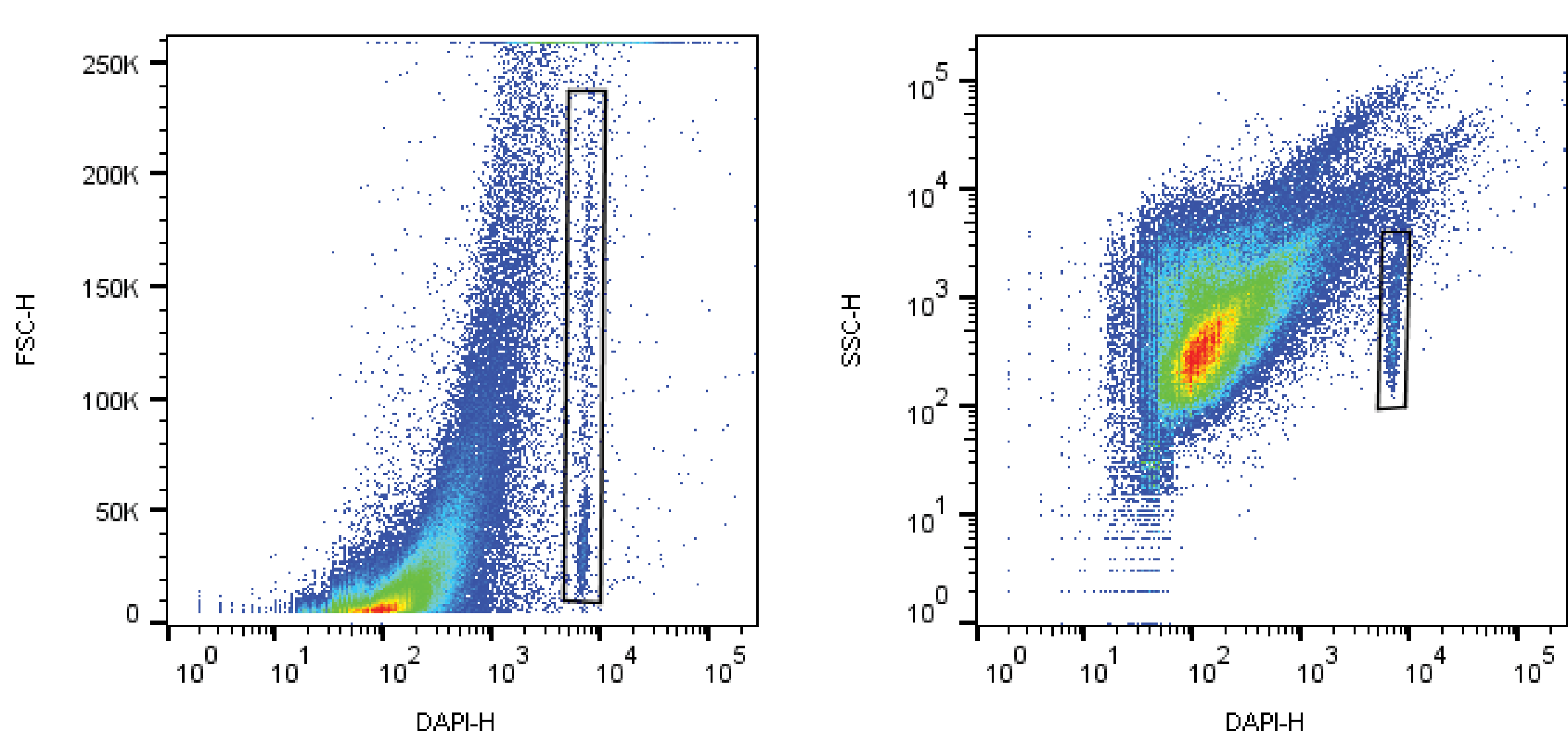

Supplement: S10 Fig — (TIFF) [file pbio.3003085.s011.tiff]

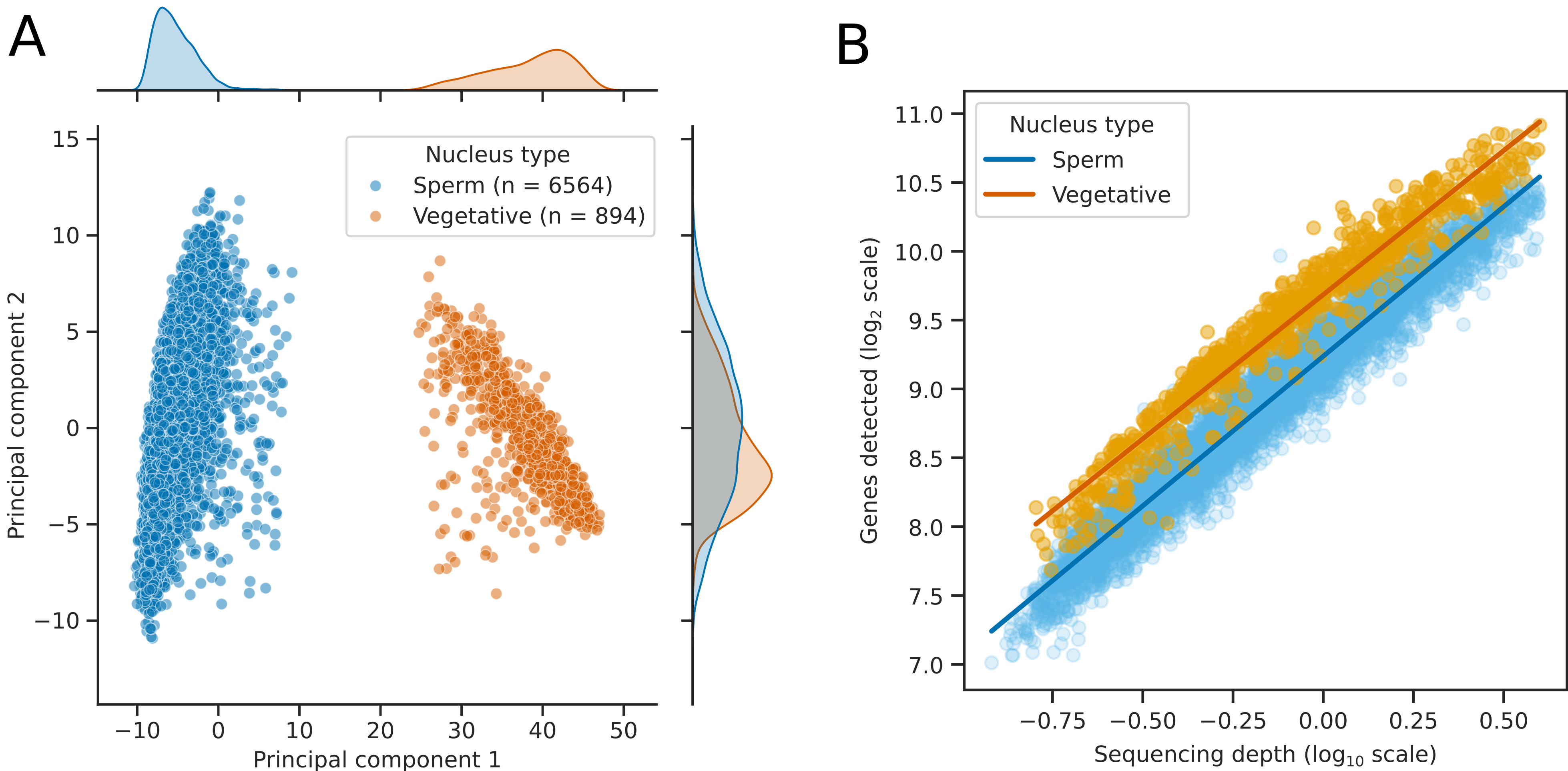

Supplement: S11 Fig — (A) Scatter plot with marginal kernel density estimates, showing the first two principal components of the expression data for 7,458 Col-0 × Db-1 pollen nuclei. Nuclei form two unequally sized clusters, mostly separated by the first principal component. (B) Log-log scale regression plot showing the relationship between per-nucleus sequencing depth and the number of detected genes, for the Col-0 × Db-1 dataset. For a given sequencing depth, more genes are detected per vegetative nucleus than per sperm nucleus, demonstrating the greater transcriptomic diversity of vegetative nuclei. The data underlying this figure can be found in dataset 4 at https://doi.org/10.5281/zenodo.14864053. (TIFF) [file pbio.3003085.s012.tiff]

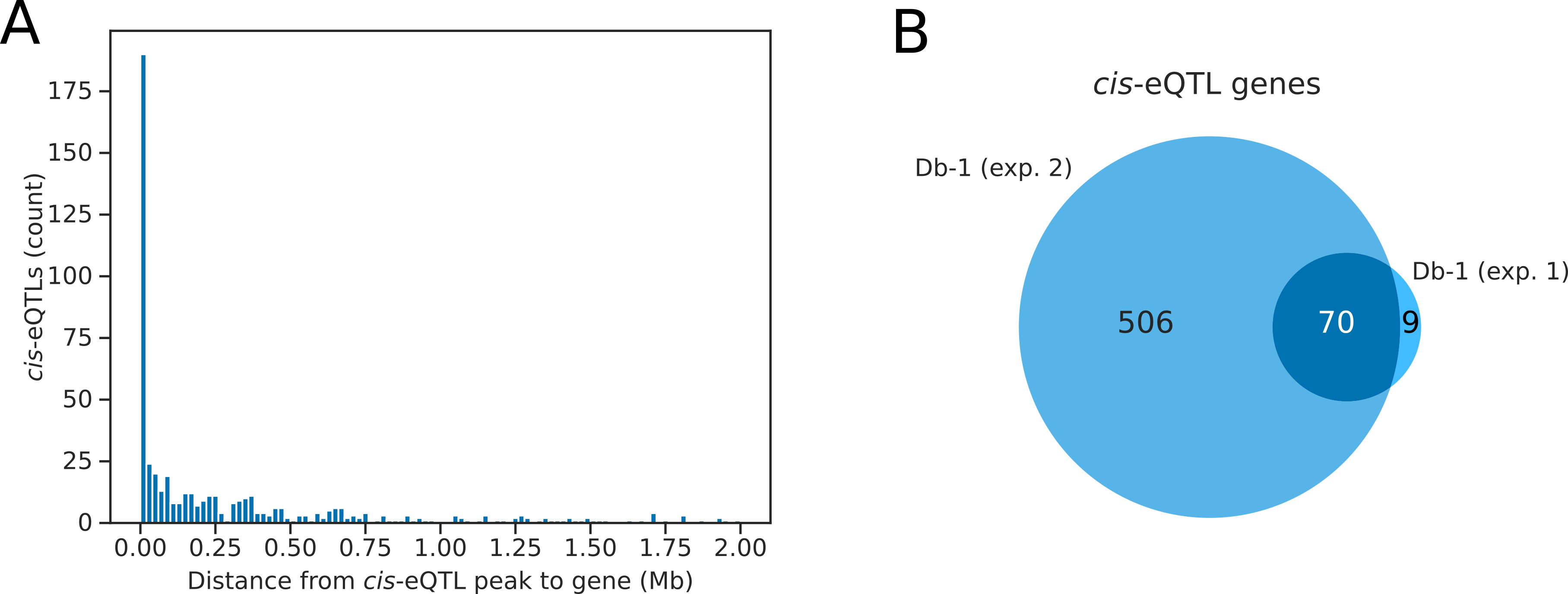

Supplement: S12 Fig — (A) Histogram showing the absolute distance from identified eQTL peaks, to the gene whose expression they correlate with, for peaks identified as being likely to be caused by cis-variants. (B) Venn diagram showing the overlap of Db-1 versus Col-0 cis-eQTLs identified in the two datasets. The data underlying this figure can be found in dataset 5 at https://doi.org/10.5281/zenodo.14864053. (TIFF) [file pbio.3003085.s013.tiff]

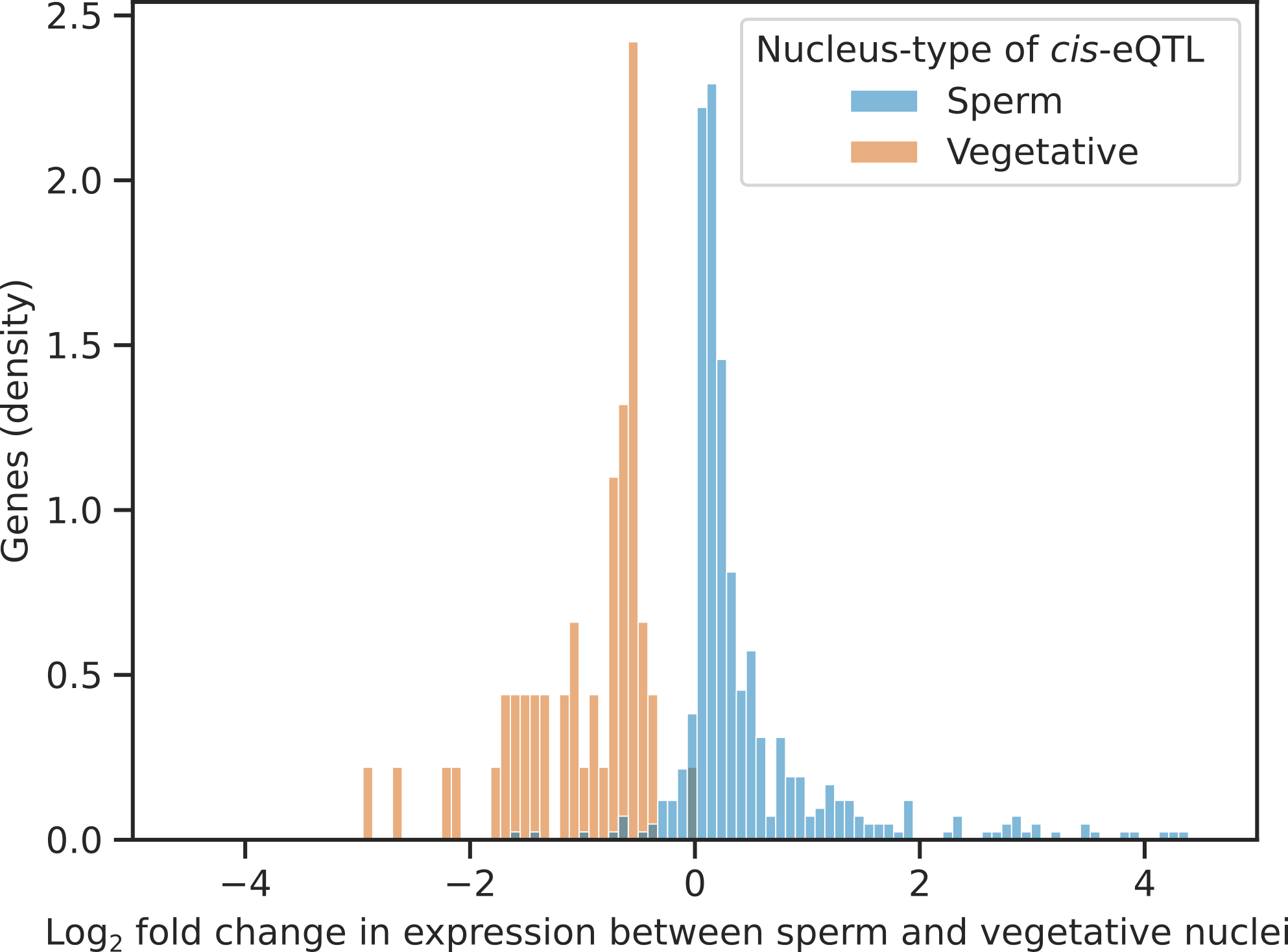

Supplement: S13 Fig — The majority of genes are only expressed in the cell-type where the eQTL is detected. The data underlying this figure can be found in dataset 5 at https://doi.org/10.5281/zenodo.14864053. (TIFF) [file pbio.3003085.s014.tiff]

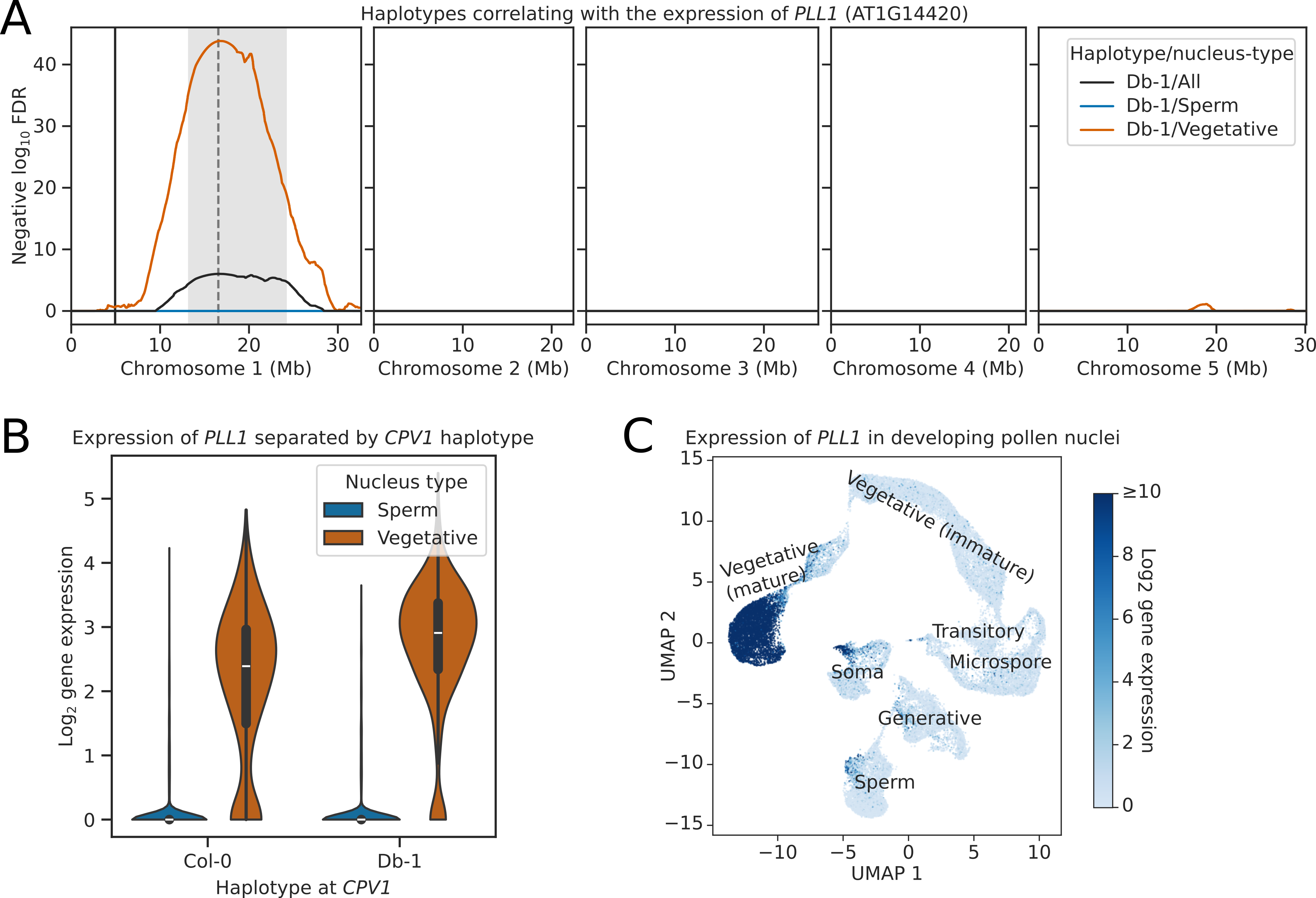

Supplement: S14 Fig — (A) eQTL plot showing the haplotypes whose inheritance correlates with the expression of the pectin lyase PLL1 (AT1G14420). eQTL peaks are shown as vertical dashed lines with 1.5 LOD drop confidence intervals shown as grey shaded regions. The location of the PLL1 gene is shown as a solid vertical black line. The black line labelled “All” shows the FDR calculated from the log ratio test of all nucleus-types combined. (B) Violinplots showing the gene expression of PLL1 in sperm and vegetative nuclei separated by the haplotype of CPV1 (Chr1: 16.5 Mb). Vegetative nuclei that inherit the Db-1 haplotype of CPV1 have higher expression of PLL1 than sister nuclei that inherit the Col-0 haplotype. (C) UMAP projection from Ichino and colleagues 2022, showing the expression of PLL1 throughout the developmental stages of the pollen. PLL1 is expressed in the mature vegetative nucleus cluster, and is absent from microspore, generative and sperm nuclei. The data underlying this figure can be found in datasets 4 and 5 at https://doi.org/10.5281/zenodo.14864053. (TIFF) [file pbio.3003085.s015.tiff]

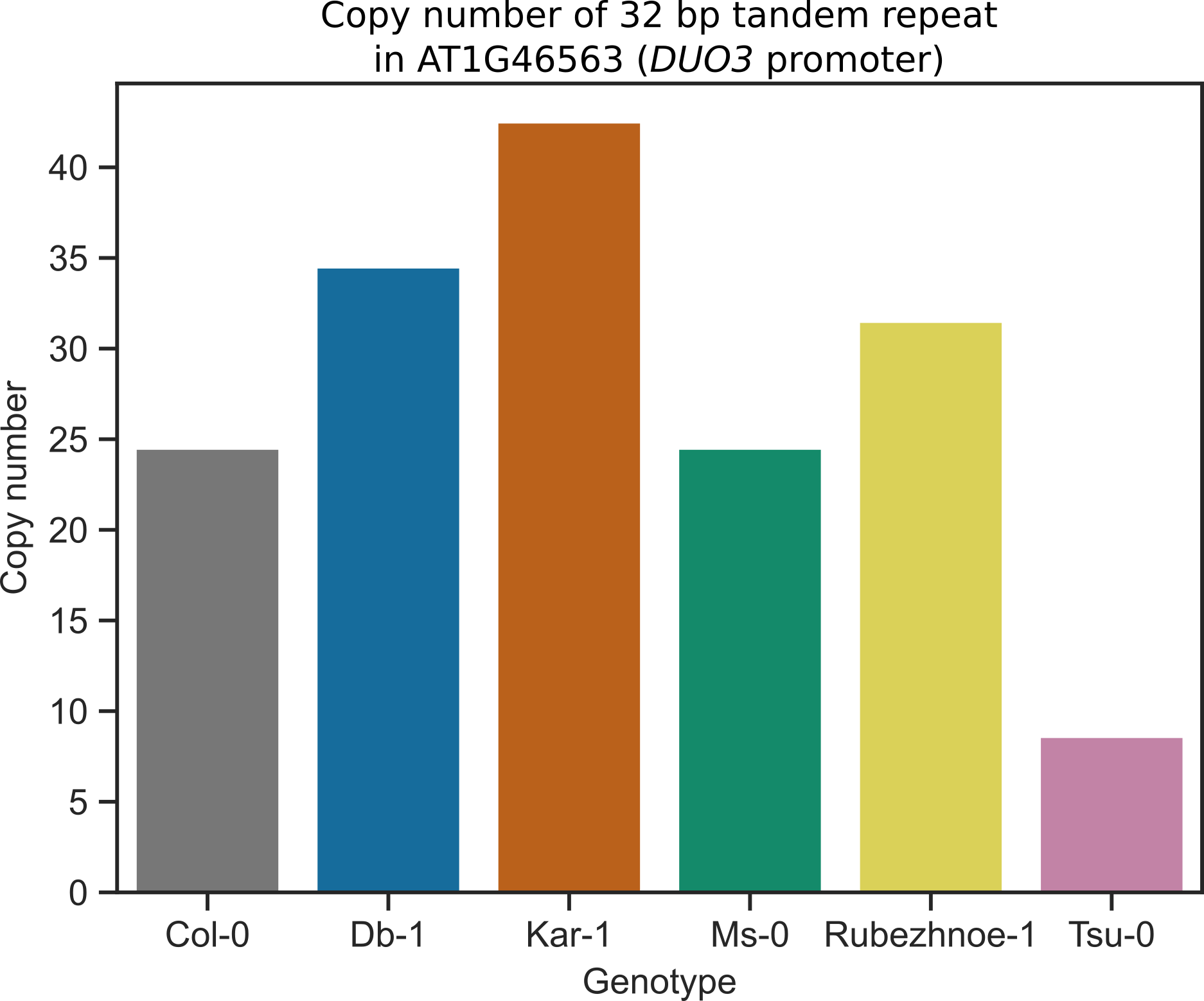

Supplement: S15 Fig — (TIFF) [file pbio.3003085.s016.tiff]

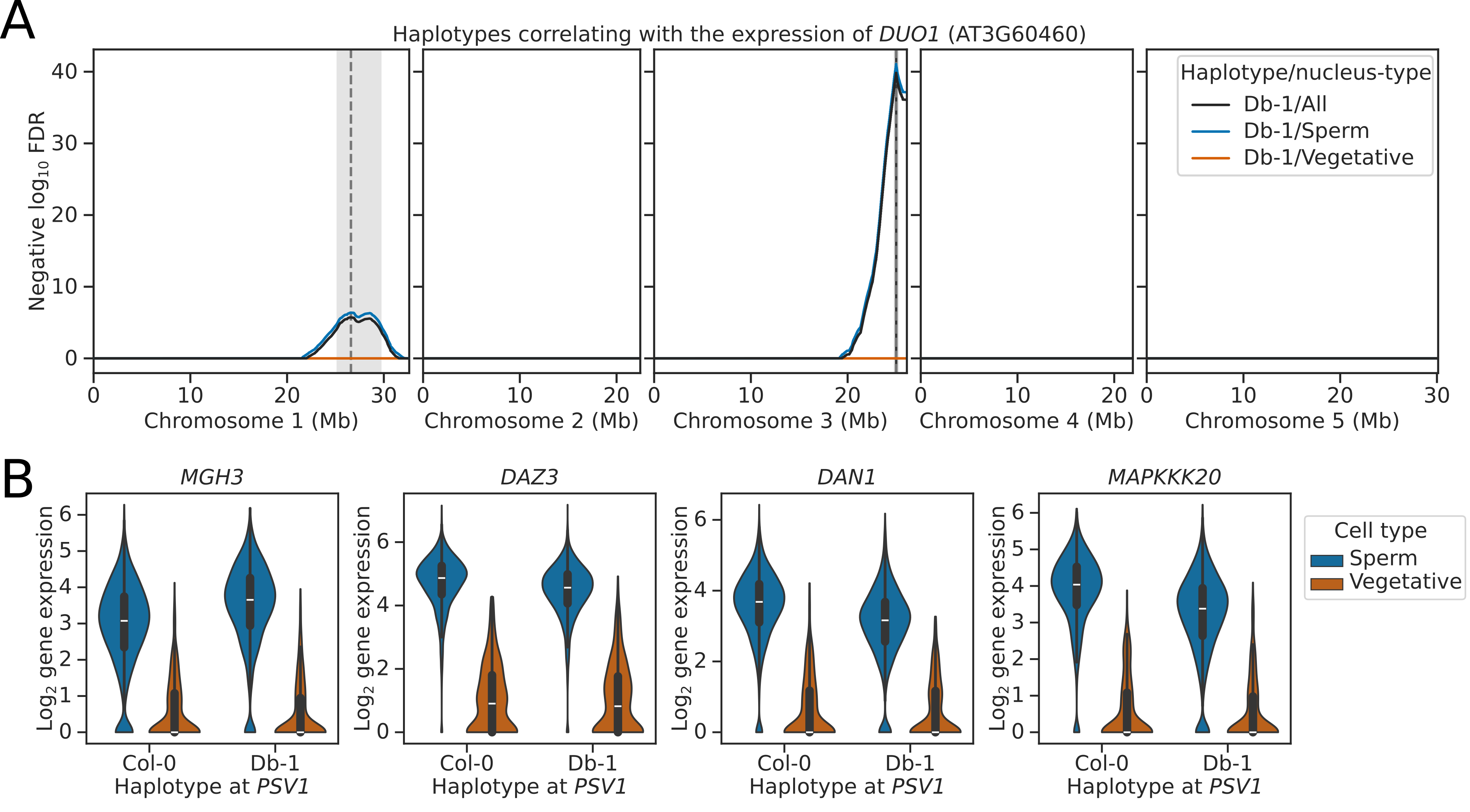

Supplement: S16 Fig — (A) eQTL peaks are shown as vertical dashed lines with 1.5 LOD drop confidence intervals shown as grey shaded regions. The location of the DUO1 gene is shown as a solid vertical black line. The black line labelled “All” shows the FDR calculated from the log ratio test of all nucleus-types combined. (B) Violinplots showing the gene expression of various characterized DUO1 targets in sperm and vegetative nuclei separated by the haplotype of PSV1. The data underlying this figure can be found in datasets 4 and 5 at https://doi.org/10.5281/zenodo.14864053. (TIFF) [file pbio.3003085.s017.tiff]
