# Supplementary material for: Scalable eQTL mapping using single-nucleus RNA-sequencing of recombined gametes from a small number of individuals
Source: PLoS Biol. 2025 Apr 25;23(4):e3003085. doi: 10.1371/journal.pbio.3003085 (PMC12119024; doi:10.1371/journal.pbio.3003085)
Supplement: Attachment — Submitted filename: 151203_1_rebuttal_3252530_spghxl.pdf [file pbio.3003085.s018.pdf]

Overall comments:

Reviewer #1:

Evidence, reproducibility and clarity

The study by Parker et al describes the innovative use of single-cell RNA sequencing to detect markers and traits of pollen. Using pollen allows detection of recombinant events allowing the ability to do quantitative trait mapping using gene expression as a trait. This led to the discovery of expected cis QTL, but there is an intriguing trans QTL as well. The trans eQTL was mapped to a candidate causal locus DUO1. This is exciting given its role in pollen development and will obviously be followed up on in future studies.

We thank the reviewer for these kind comments.

Overall, this is an exciting technological advance that will rapidly advance our ability to map pollen traits and arguably more importantly create high density recombination maps across organisms. I realize this is a proof of principle study and the questions below are intended to improve this already strong manuscript.

We agree with the reviewer that the main conceptual advance in our manuscript is the novel methodology for meiotic recombination breakpoint identification and eQTL mapping using snRNA-seq.

Significance

The overall strength is the development of a method to map recombination using single-cell genomics of pollen. The weakness is the limitation to studying pollen traits at least for now. The other weakness is helping the reader apply this to their own research questions. This is easily addressable through updating the writing in a way that is more accessible.

We thank the reviewer again for their praise of our work. Although we chose to focus on eQTL mapping in pollen at this stage, we believe that the methods we have developed could also be applied to other organisms and tissues, so long as there as genetic diversity between individual cells in the population. This was previously demonstrated using F4 segregant populations of *C. elegans* (Ben-David et al. 2021). We therefore state in the Discussion that "eQTL mapping with snRNA-seq is also possible in diploid cells from segregating populations, provided sufficiently high numbers of individuals are used as input".

The methods described in the study could be of great use to other researchers who wish to peform eQTL analyses. We therefore agree with both reviewers that the method should be made as transparent as possible in the paper. **We have therefore updated the Results, Methods and Discussion sections to address the specific points of both reviewers about clarity**.

Reviewer #2:

Evidence, reproducibility and clarity

In the manuscript by Parker et al, the authors developed a methodology to scale up eQTL studies using single-nucleus RNA-Seq of meiotic products using Arabidopsis thaliana pollen. Each pollen grain, collected from an F1 hybrid between two inbred lines, is haploid and contains a unique combination of the two parental genomes, effectively replacing the F2 population typically used in QTL studies. Single-nucleus RNA-Seq served as both phenotype and genotype, as it was used also to infer recombination profiles. In their experiments, the authors used a mixture of pollen from five different crosses - each between the reference Arabidopsis accession and another accession - for the first experiment, followed by a repeat of one of the original crosses in a second experiment to increase coverage and show reproducibility. They describe their approach to genotyping, performing the eQTL analysis, and describe statistics of the cis- and trans-eQTLs. In particular, they identify and discuss in detail a strong trans-eQTL on chromosome 1 that significantly and reproducibly associate with the expression of hundreds of genes. The likely candidate gene underlying this trans-eQTL is DUO3, a transcription factor known to play a role in pollen development, although the authors acknowledge that further experiments are needed to show that this is the causal variant.

As this is a novel methodology with the potential for widespread adoption by the community, most of my comments focus on expanding the details provided about the methodology and discussing its limitations. Additional clarity of information will help to ensure its reproducibility and wider applicability.

We agree with the reviewer that the main advance demonstrated in our study is the novel method for eQTL mapping. As noted in our response to reviewer 1, we agree that the method should be as made as clear as possible for readers, so that they can apply it to their own research questions. **We have therefore updated the Results, Methods and Discussion sections to address the specific points of both reviewers about clarity**.

Significance:

The methodology developed in this study significantly advances the feasibility of eQTL studies. The ability to easily scale up the number of individuals analyzed provides unprecedented resolution for identifying underlying alleles. In addition, because the pollen from each cross can be collected and processed together, environmental and technical variables between individuals (in this case, pollen nuclei) are tightly controlled. This advantage is underlined by the discovery of a strong trans-eQTL, which is likely to play an important role in pollen biology, and whose different alleles are spread across natural populations. I am therefore very excited about the publication of this manuscript.

We thank the reviewer for these very positive remarks.

Specific Comments:

Reviewer #1:

1. What is the average maker resolution in bp and cM? What numbers of nuclei would be needed to profile to gain single gene resolution in Arabidopsis?

For haplotyping and crossover analysis, we grouped informative reads into 25kb bins to reduce both the number of predictions required for haplotype inference, and the number of statistical tests required for eQTL mapping. We can therefore estimate the median distance between non-empty bins for each nucleus as a proxy for marker resolution resolution. We find that the median distances between non-empty bins across all nuclei are 350 kb and 500 kb for the first and second dataset respectively. **To address this question, we have updated the Results section to include some analysis of marker distributions, and added histograms of distributions as Figure 2 – figure supplement 1A.**

The question of how many nuclei would be needed to gain single gene resolution of eQTL mapping is extremely difficult to answer, as the detection and resolution of eQTLs depends on several different factors:

First and foremost, as pointed out by the reviewer, the sample size i.e. number of nuclei profiled, makes a difference to the ability to detect eQTLs.

Secondly, as in all RNA-seq experiments the effect size of the measured change in gene expression has a large impact on the ability to detect eQTLs. eQTLs causing small effect size changes will therefore require more nuclei to detect than those with large effect size changes. This likely explains why we were able to detect a larger proportion of *trans*-eQTLs in the second dataset with more nuclei compared to the first dataset, since *trans*-eQTLs tend to have smaller effect size changes.

Finally, the rate of meiotic recombination around the eQTL locus has a large impact on the resolution of the mapped eQTL. Loci in low-recombining regions, such as close to or within the centromere, will likely never be mappable to single-gene resolution, even with extremely high numbers of nuclei, due to genetic-linkage over large regions of the genome. This is exemplified by the *CPV1* locus that we mapped in the Col-0 x Db-1 cross, that maps to the centromere of Chromosome 1. Despite correlating with a relatively large effect size change in gene expression in *PLL1* (Figure 6 – figure supplement 3B), we could only map this *trans*-eQTL within a 1.5 LOD drop interval of >10 Mb. One way to address the issue of resolution caused by crossover rate could be to use mutants such as *recq4* or *figl1* which increase the rate of recombination, as was demonstrated recently (Capilla-Pérez et al. 2024).

In summary, the number of nuclei required to map an eQTL to single-gene resolution is not fixed, and depends on both the effect size of the change in expression, and the genomic location of the causal variant. **We have added a new paragraph to the Discussion to address this question and provide some potential future solutions.**

2. It would be great if the authors could add a discussion of how the resolution of mapping could be improved and what it would take to get there?

We agree this is an important question for future work. **As mentioned in response to point 1 of Reviewer #1, we have added a new paragraph to the Discussion to address this question and provide some potential future solutions.**

3. Could this approach be feasible for species where there is not a reference genome?

While this would require different methods to the ones used in our study, it is possible to identify and genetically map markers using single-cell sequencing of recombinant pollen without a reference sequence. For example, we have used single cell sequencing of hybrid pollen to assemble the genome of the pollen mother plant by providing linkage information (Sun et al. 2022; Campoy et al. 2020). We mention this in the Discussion, where we state: "it has been demonstrated that genotypes created using gamete single cell sequencing can be used to disentangle complex genome assemblies and resolve the haplotypes of polyploid species such as potato."

4. It is stated 67% are located with in 2Mb of a gene and technically that is is cis-, but there aren't really long range interactions in Arabidopsis (>30kb)...so are these really cis or trans? It might be worth considering how cis vs trans are defined. Basically, what is truly cis vs trans?

We agree with the reviewer that eQTLs that are genuinely located more than 30 kb from a gene are likely to be *trans*-eQTLs in Arabidopsis. Our reasoning for using a conservative approach to classifying eQTLs as *cis* is due to the resolution of the mapping procedure, and the expectation that the majority of identifiable eQTLs willl act in *cis,* not in *trans.* By using a conservative threshold we hope to prevent the misclassification of *cis* eQTLs as *trans.* **To better inform the reader, we provide histograms showing the distribution of distances from mapped eQTLs classified as *cis* to the gene which was used as a phenotype as Figure 3B and figure 6 – figure supplement 3A, and report the median distances from cis-eQTLs to the affected gene in the Results.**

5. What percent of the time was the actual crossover captured, if at all? Is this possible with snRNA-seq?

Assessing the accuracy of crossover mapping without ground truth information would be complex. In future we hope to use simulations and/or samples with known recombination patterns to benchmark the quality of crossover calling. This is outside the scope of the current paper. However, without knowing the true locations of crossovers we can use the probabilities produced by the rHMM to estimate 95% confidence intervals on the locations of individual crossovers. We find that the 95% confidence intervals of called crossovers follow a bi-modal distribution depending on their proximity to the centromere. For crossovers that were predicted to occur in the chromosome arms, the median 95% confidence interval size was 1.1 Mb. For crossovers that are proximal to the centromere, or where lack of markers means that the haplotype of the centromere is ambiguous, then the median 95% confidence interval size was 8.2 Mb. **We have now added information from these confidence intervals and corresponding plots showing the resolution of individual crossover calls to the Results and Figure 2 – figure supplement 1B.**

6. How many eQTL were there per locus?

We detected an average of 0.14 and 0.63 eQTLs per expressed gene in the first and second datasets, respectively. **We have added this information to the Results.**

7. How deep were the libraries sequenced and were they sequenced to saturation? In general, I did not find any sequencing summary statistics. How many reads were sequenced per library, per genotype etc, how many aligned, how many UMIs per cell, how many transcripts detected per cell. This will help give the reader a baseline for knowing what kind of quality is needed to successfully implement this strategy in their own lab.

We thank the reviewer for this important comment. **We have now added a table of sequencing statistics to the supplemental data as Supplementary file 1.**

Reviewer #2:

(1) Given the sparseness of snRNA-Seq data per nucleus/cell (inherent to single cell technologies), what happens to low expressed genes? Is any filtering done? For example, in the extreme case of a gene that is either undetected or has only a single read in some nuclei, would a sufficiently large number of nuclei theoretically allow the detection of an eQTL signal for that gene? Alternatively, does this method remain inherently blind to genes expressed below a certain threshold? Please discuss these limitations and prospects.

We indeed applied a expression filtering threshold to remove lowly expressed genes from the analysis, before performing eQTL mapping. Specifically, we removed genes which were detectably expressed (i.e. with at least one mapping read) in fewer than 5% of the cells. This is stated in the Methods section entitled "eQTL mapping analysis", and we have added clarifications to make the filtering method clearer.

(2) The methods section states that FACS sorting was used to isolate 40,000 nuclei; however, the final datasets contained only 1,394 or 7,458 nuclei. Could you provide a detailed breakdown of the losses at each step of the filtering process? As this is a key factor in the overall efficiency of the method, it would be valuable to discuss the potential for increasing throughput in future implementations. Also, how the total yield increased fivefold between repeats - could you elaborate on the factors that contributed to this improvement?

We apologise for the lack of precision in this section of the Methods in the first draft of the manuscript. **We have now updated the relevant Methods sections to include more of the concrete statistics about the preparation of nuclei samples and single-nucleus library construction.** The statistic 40,000 referred to the approximate average number of "events" that we generally aim to sort using the FACS machine. We have now removed this statistic from the Methods as it does not accurately reflect the numbers of events that were sorted in the case of these two libaries, which was in fact slightly higher**.**

For the sample used to generate the first dataset, we sorted approximately 53,000 events. Some of these may in fact represent debris, and so the true number of sorted nuclei will be less than this amount. At the time that this sample was prepared, we did not have access to a cell counter to orthogonally validate the output of the FACS machine. After sorting, the nuclei were concentrated by centrifugation before loading onto the 10x Chromium controller. Although the aim of this centrifugation step was to concentrate the nuclei, it almost certainly causes some nuclei to burst and or clump together, resulting in losses. This may explain the low recovery rate of the first dataset.

For the sample used to generate the second dataset, we sorted approximately 55,500 events and then measured the number of nuclei after sorting using a Luna FX cell counter. According to these estimates, the total number of nuclei in the sample was around 38,250 in 135µl volume. From this, 43µl containing approximately 13,150 nuclei was loaded onto the 10x Chromium controller, without centrifugation. According to the 10x Chromium next GEM single cell 3' v3.1 user guide, the reported recovery rate of singlet barcodes for this number of input cells/nuclei is around 53%, or 7000 nuclei, which is in line with the 7458 that we recovered.

The difference in yield between the first and second datasets may stem from a higher quality input sample with less debris, or from the removal of the centrifugation step from our protocol. Alternatively, it may result from advances in the 10x platform, kits and technology - the first dataset was collected in 2022, whilst the second was collected in 2024. There are many technical variables that differ between the two datasets - including the individuals who performed the experiment. This means that any prediction about the cause of the difference in yield can only really be speculative.

(3) It is unclear what happened to the vegetative nuclei during this process. While the authors attribute the differences between the first and second experiments to handling, previous studies (Schoft et al. 2015) suggest that vegetative and sperm nuclei can be distinguished in FACS analysis after DNA staining. This suggests that, for future applications, it may be possible to refine this method to specifically focus on either vegetative or sperm nuclei by adjusting the gating parameters in FACS. The inclusion of the FACS sorting graphs with the gating used as a supplementary figure would also be helpful in understanding and replicating this aspect of the methodology.

We agree with the reviewer that some of the loss of vegetative nuclei may result from the gating stragety applied during FACS. **We have added a statement clarifying this to the Results.** We are now working with fluorescent reporter lines that distinguish the sperm and vegetative nuclei, to determine more appropriate gating strategies for vegetative nuclei, and hope to improve the recovery of these nuclei in the future. **As suggested by the reviewer, we have added the gating strategies of the two FACS experiments as supplementary Figure 1 – figure supplement 1, and Figure 6 - figure supplement 1.**

(4) For the benefit of future users, it would be helpful to discuss the following points: (1) Would you recommend using a combination of several parents, as in the first experiment, or limiting the analysis to two parents? (e.g. it seems some of the recombination patterns could not be called due to the mix of five crosses) What are the advantages and disadvantages of each approach? (2) How should one estimate the optimal number of nuclei/cells to use? Can you downsample the nuclei and see the effects of lower numbers on identified eQTLs? (3) Given that different single-cell RNA-Seq protocols involve trade-offs between the number of cells and the number of UMIs per cell in relation to cost, what strategy would you recommend to users to optimize their experiments?

The design of experiments depends on the research goals and budget of the project. However, we do have some recommendations for mixing of different genotypes – namely, that care must be taken to select genotypes to pool which are sufficiently genetically distinct, since genetically similar genotypes will be harder to distiguish and demultiplex correctly. Where

possible, pooling is invaluable as a way to control for technical variation. When pooling is not possible due to genetically indistiguishable genotypes, then experimental design and randomisation must be considered carefully to prevent confounding. **We have added some recommendations in this regard to the Discussion.**

We addressed the question of number of nuclei on the detection and resolution of eQTLs in response to Reviewer #1 point 1. **We have added a new section to the Discussion to address this question.** In general it is hard to perform power analyses prior to conducting transcriptomic experiments, because the effect size of gene expression changes of interest are generally not known in advance. More general investigations of the trade off between number of cells and number of UMIs in single-nucleus sequencing experiments have been thoroughly investigated by other groups (Svensson et al. 2017; Mandric et al. 2020).

(5) It is not entirely clear which exact linear model was used for the association study. Specifically: (1) How were the five parents included in the model? (2) Why was it necessary to correct for population structure in this case, given the controlled crosses? (3) How was technical variation accounted for, and how were principal components derived and incorporated into the analysis? (4) What is meant by "cell type control" and were the few VN included in the analysis? To ensure clarity and reproducibility, it would be helpful to provide more detailed explanations and to explicitly state the linear equation used for fitting.

We apologise for the confusion as to the linear model used. **We have now updated the Methods section to make this clearer and explictly included the formula of the linear model.** For an experiment containing N pooled hybrids, for each barcode the predicted genotype of Parent 2 (as the first parent was always Col-0) was included in the model using (N - 1) dummy variables, and the predicted haplotype as a set of N continuous variables (with zero representing either the Col-0 allele, and one representing the Parent 2 allele). Despite the controlled crosses, the genotype of Parent 2 has to be controlled in the equation because the mix of five hybrids is similar to population structure, i.e. haplotypes that are only found in Kar-0 are reasonably correlated in the dataset, even when these haplotypes are completely unlinked, because only nuclei from the Col-0 x Kar-0 cross can share them. Controlling for Parental genotype prevents the detection of these as spurious correlations.

In the initial models, cell type was not explicitly controlled but was captured primarily by principal component 1 of the PCA, which was used as a covariate. The vegetative nuclei were not excluded from the analysis, but likely did not contribute strongly due to their limited number. In the models where haplotype x cell type was modelled, cell type cluster was also explicitly used as a covariate. **We have updated the Methods to make these points clearer.**

(6) To my understanding, utilizing the 10X platform with the ATAC-Seq option could provide a much more accurate recombination map. This approach would allow the inclusion of SNP information from non-transcribed regions and lowly expressed genes, which are often missed with current methods. Perhaps this is a useful consideration to add to the discussion as a potential improvement for future studies?

We indeed already mention the possibility of using single nucleus ATAC-seq for crossover analysis in the Discussion section. In addition to the theoretical improvements to the recombination mapping mentioned by the reviewer, snATAC-seq could also be used for molecular QTL mapping, to identify so-called “chromatin-accessibility” or caQTLs. A limitation

however, would be reduced power for QTL mapping, due to the noisier nature of the molecular phenotype: on a single cell level, chromatin accessibility is an inherently binary phenotype, i.e. either a read is identified in a peak or it is no, but the absence of a read in a peak is not necessarily evidence that the region is closed in that cell, due to sparsity and high levels of dropouts.

(7) Could you clarify how the five parents from the larger panel referred to in the paper were selected? What criteria were used and how might this selection affect the results or applicability of the methodology?

The selection of the five parents in the larger panel was determined by practical considerations. We performed a large number of crosses to Col-0 using available accessions for which there were available genome assemblies, that also had similar flowering times and vernalisation requirements. Only a proportion of these crosses were successful. From the remaining hybrids we then selected five which represented geographical diversity, and that we also felt should be genetically distinct enough to demultiplex using variants. We do not see how the selection could have affected the applicability or generalisability of the methodology.

(8) In most of the analyses using the mixture of pollen from five crosses, the data are predominantly from sperm cells. It is not clear whether VN data were explicitly removed, or whether VN data were included at any stage of the analysis of the first experiment. Given that almost all the data are from sperm nuclei, it might be more accurate to consistently refer to results from this first dataset as "sperm" rather than using terms such as "pollen nuclei" or "pollen gene expression".

To be clear, vegetative nuclei were not specifically removed from any analyses, however due to their low number in the first dataset we did not attempt to map vegetative nucleus specific eQTLs. **We have updated the Methods to make this point clearer.**

(9) As many of the cis-eQTLs result from structural variations, in some cases related to the presence or absence of the gene in question itself, and given that expression quantification is performed relative to the reference genome, could you provide statistics on the following? Specifically, how often is gene expression higher when the haplotype is from the reference parent compared to the other parents? It would be helpful to break this down into different categories of identified eQTLs (cis vs. trans, and within cis, structural variants vs. other variations). This analysis would provide an estimate of the reference bias inherent in this quantification approach.

The reviewer is correct that there will be some reference bias when it comes eQTLs caused specifically by gene presence-absence variation, because only genes which are present in the Araport11 annotation are tested for eQTLs. This means that genes which are present in Col-0 but absent in other accessions can be identified, whereas genes that are absent in Col-0 but present in other accessions cannot. **We have added a caveating statement to the Discussion to make this clear.** In aggregate, however, we do not see a strong bias gene expression change direction amongst genes with *cis*-eQTLs. In the Col-0 x Db-1 cross, for example, we see that 51.3% of genes with *cis*-eQTLs have higher expression in Db-1 than in Col-0 (χ^2 p = 0.53).

(10) The authors discuss some examples of PSV1 genes in the context of the cell cycle. Could you provide additional statistical measures to support these findings, such as GO enrichment analyses for these genes?

We performed hypergeometric test analysis to test the enrichment of cell cycle factors reported in Supplementary File IX of Van Leene et al., 2010, amongst the genes with a *PSV1 trans*-eQTL (Van Leene et al. 2010). Of the 501 unique genes in this list, 112 were expressed in our snRNA-seq dataset to a sufficient level to be tested for eQTLs. Of these, 33 had a mappable *trans*-eQTL at the *PSV1* locus. This represents a statistically significant enrichment of genes annotated as involved in the cell cycle ($p = 0.032$). **We have added this analysis to the Results section.**

(11) The crosses were grown under slightly different conditions. While I do not suggest repeating the experiment, especially as the main PSV1 result was reproducible, it would be useful to determine whether pollen collected from these two conditions show similar gene expression patterns, even in bulk. This analysis could shed light on whether pollen gene expression is relatively insensitive to these environmental variations, and whether the second trans-eQTL CPV1 is specific to plants grown in 18C.

We agree that the combination of pollen from two temperature conditions in the second experiment is not an ideally designed experiment. We had expected that differences caused by temperature variation would be identifiable as prinicipal components in the single nucleus sequencing data, however this was not obviously the case. Unfortunately, we do not have remaining pollen material from these samples to perform bulk RNA sequencing or qPCR analysis. Although we cannot rule out a temperature effect explaining *CPV1,* we believe that the most likely explanation for why this was not identified in the first dataset is that it is specific to vegetative nuclei, of which there were very few in the first dataset, and also perhaps to the Col-0 x Db-1 comparison (unlike *PSV1* which appears to be shared in at least Col-0 x Db-1 and Col-0 x Rubezhnoe-1 sperm nuclei), meaning that there was much less power to detect it.

(12) "Approximately 87.4% of cis-eQTLs were specific to sperm nuclei, likely reflecting the greater statistical power for sperm. " - This claim can be checked by downsampling the number of nuclei used in the sperm analysis to the same number as in the VN.

In principal downsampling analysis could be used to test this hypothesis, however we feel it would unnecessarily add a confusing element to the manuscript. In future we will consider performing a thorough benchmarking and power analysis of the eQTL method, however we feel this is currently out of the scope of this "proof of principle" manuscript. **We have instead opted to remove this speculative statement from the manuscript.**

(13) "This suggests either that CPV1 affects different sets of genes in sperm and vegetative nuclei, or possibly that there are two independent variants underlying CPV1 which affect sperm and vegetative gene expression respectively" - As done with the genes affected by PSV1, can you use the data from Ichino et al. to show in which cell types the genes affected by CPV1 alleles are expressed?

As requested, we have added a UMAP-plot for the example *CPV1 trans*-eQTL affected gene *PLL1* showing its expression in mature vegetative nuclei, as Figure 6 – figure supplement 4C.

(14) "DUO3 is expressed at a low level however, and is only detectable in 14.1% of Col-0 × Db-1 nuclei," - Can you pseudo-bulk the nuclei according to the haplotype of PSV1 to get a better estimate of DUO3 expression levels in each allele? Or would this be equivalent to the linear model currently used?

As the reviewer themself states, this approach would be somewhat equivalent to the linear model approach currently used, with the downside of not being able to control for other factors such as cell type or principal components.

(15) "studies suggesting that Arabidopsis pollen is 2C (Friedman, 1999", - To my understanding Friedman 1999 reports that sperm are stopped in the middle of S phase and thus not with 2C genomic content.

Thank you for this correction. **We have now updated this statement to read: "studies suggesting that Arabidopsis pollen has a DNA content greater than 1C".**

(16) Could you include a heatmap showing the called recombination profiles, similar to the background colors in Fig. 2C, for all nuclei arranged by their snRNA-Seq coverage? This would provide a clearer visualization of the data distribution and the relationship between coverage and accuracy of recombination patterns calling.

We have added a heatmap as requested as Figure 2 – figure supplement 2.

(17) Some figures (& captions) are missing important details or could benefit from clarification: (1) In Fig. 2B, it is not defined what the orange represents. (2) In Figs. 3A and 6A, the size of the dots is not defined. (3) Aesthetic note: In Fig. 3B, the same colors as in Fig. 3A are used, although they represent different categories. I suggest modifying the color scheme for better clarity. (4) In Fig. 4A, it is unclear what "All" refers to. (5) In the caption for Fig. 4, panels (E) and (F) are mistakenly labeled as (B) and (C).

We thank the reviewer for these important suggestions. **We address them here point-by-point:**

(1) In the lower panels of figure 2B, the blue and orange lines represent the marker read distributions supporting the Col-0 and parent 2 haplotypes, respectively. **We have updated the figure legend to clarify this.**
(2) The sizes of the points in figures 3A and 6A are proportional to the LOD score of the eQTLs. **We have updated the figure legends to make this clear.**
(3) **We have altered the colours of Figure 3B (now also relabeled as Figure 3C).**
(4) In Figure 4A, as well as Figure 4D, Figure 4 – figure supplement 1A and Figure 5 – figure supplement 1A, the black line "All" represents the negative log10 FDR from the log ratio test of all haplotypes, i.e. whether there is an overall association between a locus

and the expression of the target gene, in any or all genotypes compared to Col-0. **To make this clearer, we have updated the figure legends.**

(5) We thank the reviewer for spotting this oversight. **We have now corrected the labelling of the figure legends.**

Ben-David, Eyal, James Boocock, Longhua Guo, Stefan Zdraljevic, Joshua S. Bloom, and Leonid Kruglyak. 2021. "Whole-Organism EQTL Mapping at Cellular Resolution with Single-Cell Sequencing." *ELife* 10 (March). https://doi.org/10.7554/eLife.65857.

Campoy, José A., Hequan Sun, Manish Goel, Wen-Biao Jiao, Kat Folz-Donahue, Nan Wang, Manuel Rubio, et al. 2020. "Gamete Binning: Chromosome-Level and Haplotype-Resolved Genome Assembly Enabled by High-Throughput Single-Cell Sequencing of Gamete Genomes." *Genome Biology* 21 (1): 306.

Capilla-Pérez, Laia, Victor Solier, Elodie Gilbault, Qichao Lian, Manish Goel, Bruno Huettel, Joost J. B. Keurentjes, Olivier Loudet, and Raphael Mercier. 2024. "Enhanced Recombination Empowers the Detection and Mapping of Quantitative Trait Loci." *Communications Biology* 7 (1): 829.

Mandric, Igor, Tommer Schwarz, Arunabha Majumdar, Kangcheng Hou, Leah Briscoe, Richard Perez, Meena Subramaniam, et al. 2020. "Optimized Design of Single-Cell RNA Sequencing Experiments for Cell-Type-Specific EQTL Analysis." *Nature Communications* 11 (1): 5504.

Schoft, Vera K., Nina Chumak, János Bindics, Lucyna Slusarz, David Twell, Claudia Köhler, and Hisashi Tamaru. 2015. "SYBR Green-Activated Sorting of Arabidopsis Pollen Nuclei Based on Different DNA/RNA Content." *Plant Reproduction* 28 (1): 61–72.

Sun, Hequan, Wen-Biao Jiao, Kristin Krause, José A. Campoy, Manish Goel, Kat Folz-Donahue, Christian Kukat, Bruno Huettel, and Korbinian Schneeberger. 2022. "Chromosome-Scale and Haplotype-Resolved Genome Assembly of a Tetraploid Potato Cultivar." *Nature Genetics* 54 (3): 342–48.

Svensson, Valentine, Kedar Nath Natarajan, Lam-Ha Ly, Ricardo J. Miragaia, Charlotte Labalette, Iain C. Macaulay, Ana Cvejic, and Sarah A. Teichmann. 2017. "Power Analysis of Single-Cell RNA-Sequencing Experiments." *Nature Methods* 14 (4): 381–87.

Van Leene, Jelle, Jens Hollunder, Dominique Eeckhout, Geert Persiau, Eveline Van De Slijke, Hilde Stals, Gert Van Isterdael, et al. 2010. "Targeted Interactomics Reveals a Complex Core Cell Cycle Machinery in Arabidopsis Thaliana." *Molecular Systems Biology* 6 (1): 397.
